# Predictive value of circulating interleukin-6 and heart-type fatty acid binding protein for three months clinical outcome in acute cerebral infarction: multiple blood markers profiling study

**DOI:** 10.1186/cc12564

**Published:** 2013-03-16

**Authors:** So-Young Park, Jinkwon Kim, Ok-Joon Kim, Jin-Kyeoung Kim, Jihwan Song, Dong-Ah Shin, Seung-Hun Oh

**Affiliations:** 1 Department of Neurology, CHA Bundang Medical Center, CHA University, 351 Yatap-dong, Seongnam, 463-712, Republic of Korea; 2 Department of Pharmacy, CHA University, 222 Yatap-dong, Seongnam, 463-070, Republic of Korea; 3 Department of Biomedical Science, CHA Stem Cell Institute, CHA University, 606-16 Yeoksam-dong, Seoul, 135-081, Republic of Korea; 4 Department of Neurosurgery, Yonsei University College of Medicine, 134 Sinchon-dong, Seoul, 120-752, Republic of Korea

## Abstract

**Introduction:**

There is no single blood marker for predicting the prognosis in ischemic stroke. A combination of multiple blood markers may enhance the ability to predict long-term outcome following ischemic stroke.

**Methods:**

Blood concentrations of neuronal markers (neuron-specific enolase, visinin-like protein 1, heart type fatty acid binding protein (hFABP) and neuroglobin), astroglial markers (S100B and glial fibrillary acidic protein), inflammatory markers (IL-6, TNF-α, and C-reactive protein), blood-brain barrier marker (matrix metalloproteinase 9), and haemostatic markers (D-dimer and PAI-1) were measured within 24 hours after stroke onset. The discrimination and reclassification for favorable and poor outcome were compared after adding individual or a combination of blood markers to the clinical model of stroke outcome.

**Results:**

In multivariate analysis, natural log-transformed (_log_) IL-6 (odds ratio (OR): 1.75, 95% CI: 1.25 to 2.25, *P *= 0.001) and _log_hFABP (OR: 3.23, 95% CI: 1.44 to 7.27, *P *= 0.005) were independently associated with poor outcome. The addition of a single blood marker to the clinical model did not improve the discriminating ability of the clinical model of stroke outcome. However, the addition of the combination of _log_IL-6 and _log_hFABP to the clinical model showed improved discrimination (area under receiver operating characteristic (AUROC) curve: 0.939 versus 0.910, *P *= 0.03) and reclassification performance (net reclassification improvement index: 0.18, *P *= 0.005).

**Conclusions:**

A combination of circulating IL-6 and hFABP level has an additive clinical value for the prediction of stroke outcome.

## Introduction

The early prediction of long-term clinical outcome following ischemic stroke is important because of the high morbidity and mortality associated with stroke. Intravenous tissue plasminogen activator (tPA) is the only effective treatment for ischemic stroke, but less than 15% of all patients are eligible for thrombolytic therapy due to its narrow therapeutic window [1]. In patients who were not indicated for thrombolytic therapy, early initiation of treatment to reduce secondary neuronal damage is required for a favorable outcome. Age and initial clinical severity, represented by the National Institutes of Health Stroke Scale (NIHSS) score, are established clinical predictors for long-term outcome [2]. Brain imaging is a powerful diagnostic tool to determine the extent of neuronal injury which is associated with long-term stroke outcome. However, brain imaging has several limitations with regard to feasibility and cost-effectiveness.

Blood biomarkers can be a complementary tool for diagnosis [3], predicting prognosis [4,5] and therapeutic monitoring of novel treatments in ischemic stroke [6]. Blood markers of neuronal injury, astroglial injury, inflammatory mediators, and/or thrombotic/haemostatic proteins are candidate blood markers for early stroke recurrence or long-term clinical outcome in previous case-control studies [7-11]. For the clinical application of biomarkers for stroke prognosis, candidate blood markers must satisfy the proposed criteria for an ideal blood marker [4,12]; 1) the marker has a statistically independent association with outcome after adjusting for clinical covariates; 2) the marker improves the discriminating ability of the established clinical model; and 3) the marker can reclassify patients at low or high risk for poor outcome across clinically relevant thresholds of predicted probabilities of stroke outcome.

Although many blood markers have been investigated to date, no single blood marker has been proven to be clinically useful to predict stroke outcome [12]. The reason is that most of the previous studies measured a single blood marker in a case-control design with variable time points of sampling. Profiling studies of multiple markers, the mechanisms of which are differentially associated with stroke pathophysiology, have tried to overcome the limitation of a single biomarker for stroke diagnosis [13] or outcome [4,5,10]. Several panels of inflammatory and haemostatic markers, such as IL-6, N-terminal pro-brain natriuretic peptide, C-reactive protein (CRP), fibrinogen, and/or D-dimer, have been suggested to be associated with poor clinical outcome and death [4,5,10]. However, whether the addition of blood biomarkers improves the predictability of the clinical predictors for stroke outcome remains controversial [4,5,10]. Therefore, clinically applicable panels of blood markers should be evaluated in subsequent studies.

The aim of the present study is to measure the blood concentration of multiple blood markers in stroke patients and investigate the clinically available blood markers for prediction of the long-term clinical outcome.

## Materials and methods

### Study subjects

We received informed consent from all patients or their families, and the Institutional Review Board at CHA Bundang Medical Center approved the study protocol (IRB protocol no: 2008-034). Among the 353 patients with clinical presentation of stroke who were admitted to the CHA Bundang Medical Center between January 2009 and June 2010, we included only patients who met the following inclusion criteria: (1) symptom onset between 6 and 24 hours prior to blood sampling, (2) first-ever-stroke without any previous history of neurological disease, (3) adequate access to patient's information, and (4) agreement to participate in the study protocol. We excluded patients with (1) intravenous or intra-arterial thrombolytic therapy (*n *= 26), (2) interventional treatment (*n *= 6), (3) neurological symptoms that resolved within 24 hours (*n *= 21), (4) history of malignancy (*n *= 11), (5) evidence of acute systemic infection (for example, fever, pneumonia, urinary tract infection) at the time of blood sampling (*n *= 8), (6) cerebral hemorrhage and head trauma (*n *= 14), or (7) refusal to participate in the study protocol (*n *= 83). Among the 184 patients, 9 patients were later excluded because the blood samples showed poor quality or hemolysis during preparation and were discarded. Therefore, a total of 175 cases were included in the multiple blood marker study (age, 66 ± 11 years, 54% male).

### Assessment of clinical parameters

Ischemic stroke is defined as a clinically definite stroke in a patient whose brain imaging shows a positive lesion on brain magnetic resonance imaging (MRI) or vascular insufficiency relevant to clinical symptoms. At 72 hours after the stroke, all of the patients underwent diffusion weighed imaging (DWI) of the brain MRI to examine the presence of acute infarction. Additional cerebral angiography (one or combined computed tomography (CT)-, MR and/or conventional cerebral angiography) was performed to detect vascular occlusion in all of the patients during hospitalization. All stroke patients underwent electrocardiography and cardiac enzyme (troponin and CK-MB) evaluation at admission, and none had evidence of concurrent acute coronary syndrome. Additional cardiac tests included trans-thoracic echocardiogram (95%), trans-esophageal echocardiogram (74%), and 24-hour electrocardiogram monitoring (77%).

Vascular risk factors were determined as positive past history, current treatment and positive laboratory findings, including systolic and diastolic blood pressure (SBP and DBP), blood tests for fasting glucose, creatinine, and lipid profile. Hypertension was defined as a high baseline blood pressure (systolic ≥ 140 mm Hg or diastolic ≥ 90 mm Hg) or a history of antihypertensive medication. Diabetes mellitus (DM) was defined as fasting plasma glucose of ≥ 126 mg/dL or a history of insulin or oral hypoglycemic therapy. Smoking was defined as current smokers at the time of examination. Hyperlipidemia was defined as fasting serum total cholesterol of ≥ 220 mg/dL or a history of treatment with a statin. Cardiac disease included a previous history or evidence of valvular heart disease, ischemic heart disease, atrial fibrillation (Afib) and heart failure. All of the stroke patients underwent standardized neurological examination by experienced neurologists during hospitalization. Neurological severity was quantified using NIHSS scores from admission to discharge. Long-term clinical outcome was determined by the use of a modified Rankin scale score at three months (3m-mRS) after symptom onset by the same neurologists who were blinded to laboratory results. The favorable outcome group was defined as patients with 3m-mRS scores of 0 to 2 (*n *= 111), and the poor outcome group was defined as patients with 3m-mRS scores ≥ 3 (*n *= 64). The mechanism of stroke was evaluated according to Trial of Org10172 in Acute Stroke Treatment (TOAST) criteria [14].

### Measurement of plasma concentration of biomarkers and infarct volume

Whole blood (10cc) was drawn on arrival at the emergency department using a tube containing ethylenediaminetetraacetic acid (EDTA) or citrate. The samples were immediately delivered to the laboratory. Plasma and serum were quickly isolated from whole blood via centrifuge (3,000 g for 15 minutes), and the samples were stored at -80°C for later analysis. In laboratories blinded to clinical information, a total of 12 panel markers, including: (1) neuronal markers: neuron-specific enolase (NSE), neuroglobin (Ngb), heart-type fatty acid binding protein (hFABP) and visinin-like protein 1 (VSNL-1); (2) astroglial markers: S100B and glial fibrillary acidic protein (GFAP); (3) inflammatory markers: C-reactive protein (CRP), IL-6 and TNF-α; (4) blood-brain barrier marker: matrix metalloproteinase-9 (MMP-9); and (5) haemostatic markers: D-dimer and plasminogen activator inhibitor-1 (PAI-1), were measured using commercially available ELISA kits according to the manufacturer's protocols. We selected blood markers that were plausibly associated with the pathophysiology of ischemic stroke, based on previous experimental and clinical studies. Detailed methods for the measurement of individual blood markers are described in Additional file 1.

Infarct volume was assessed using 72-hour DWI of hyperintense lesions. The infarct size at each slice was initially measured using a semi-computerized, intensity-threshold technique (Image J software, NIH, Bethesda, MA, USA). The infarct volume was calculated as the infarct size multiplied by the inter-slice thickness (6 mm), and the total infarct volume was determined by summating the infarct volumes at each slice.

### Statistical analysis

The categorical data (sex and presence of vascular risk factors) were compared between two groups using the χ2 test. Continuous data (age, SBP, DBP, routine laboratory data, and blood markers) were compared between two groups using the Student's t-test or a Mann-Whitney U test if the variables were not matched to a standard normal distribution. All tested blood markers showed highly skewed data (Kolmogorov-Smirov test < 0.02) and were natural log-transformed (+1 if zero value is present). As most stroke patients showed negative GFAP (71.4%) and VSNL-1 (93.1%) in the peripheral blood at this time point, these markers are regarded as binary variables (positive or negative). Data of continuous variables were expressed as mean ± standard deviation or median with IQR. The relationship between blood markers and infarct volume was evaluated with Spearman's correlation coefficient test.

For independent association between individual blood markers and stroke outcome, each natural log-transformed (_log_) blood marker was entered into a separate model after adjusting for age and initial NIHSS score. We constructed a baseline clinical model including age and initial NIHSS score for stroke outcome using logistic regression analyses [15]. Age and initial NIHSS score showed a strong association with stroke outcome after adjusting for other clinical covariates, such as vascular risk factors, Afib and white blood cell (WBC) count in multivariate analysis (*P *< 0.01). Vascular risk factors, Afib and WBC count were not statistically significant after adjusting for age and initial NIHSS score (all *P *> 0.05). Although infarct volume and TOAST subtype were associated with stroke outcome, we excluded these variables as clinical covariates because: 1) these variables could not be obtained within 24 hours after stroke onset; 2) multi-collinearity was suspected between infarct volume and NIHSS score (*r *= 0.65, *P *< 0.0001); and (3) the statistical power of these variables was weaker than the NIHSS score for prediction of stroke outcome. A *P *value of < 0.005 for each blood marker was regarded as significant for multiple comparison hypotheses (comparison of 12 blood markers in this study).

To examine whether the addition of blood markers improved the predictability of the baseline clinical model for stroke outcome, we conducted multivariate logistic regression analysis by entering individual or a combined set of _log _IL-6 and _log _hFABP into the baseline clinical model. The changes of -2log likelihood ratio and Hosmer-Lemeshow's model were compared among the models. The ability of the model to discriminate between favorable and poor outcome was assessed by receiver operating characteristic (ROC) curve analysis in each model. The differences in area under the curve (AUC) between the blood marker model and baseline clinical model were tested using the Delong method with regard to whether the addition of individual or combined blood markers improved the discrimination between the favorable and poor outcome groups. Additionally, we calculated the net reclassification improvement (NRI) index and integrated discrimination index (IDI) with the clinically relevant thresholds of the predicted probability of baseline clinical model as proposed by a previous study [4]: 0.10 (very low risk), 0.50 (intermediate risk), and 0.90 (very high risk). We determined these thresholds because we examined whether the addition of blood markers can reclassify the very low-risk and very high-risk groups for poor outcome who need conservative care or aggressive treatment. Values less than 0.05 were considered significant associations. The statistical analyses were performed using SPSS version 18.0 (SPSS, Chicago, IL, USA), MedCalc version 11.3 (Mariakerke, Belgium), and the R package for Windows (version 2.12.2).

## Results

The demographic and laboratory characteristics between the favorable and poor outcome groups are described in Table 1. The poor outcome group was older, had a higher WBC count and had a higher prevalence of Afib than the favorable outcome group. The poor outcome group had a higher median initial NIHSS score and larger median DWI volume than the favorable outcome group. The univariate logistic regression model indicates that age (OR: 1.58 (per 10 years), 95% CI: 1.17 to 2.12, *P *= 0.002), initial NIHSS score (OR: 4.20 (per 3 points), 95% CI: 2.70 to 6.52, *P *< 0.001), Afib (OR: 4.29, 95% CI: 2.17 to 8.48, *P *< 0.001) and WBC count (adjusted OR: 1.11 (per 10^3^/μl), 95% CI: 1.03 to 1.19, *P *= 0.005) were associated with poor prognosis. TOAST classification, especially small vessel disease subtype and small infarct volume were associated with favorable outcome (*P *< 0.01). In the multivariate analysis of clinical variables, only age and initial NIHSS showed a strong association with poor stroke outcome (all *P *< 0.01), with the disappearance of significance for Afib and WBC count (*P *> 0.05). Among nine deceased patients (3m-mRS = 6), six died from the stroke itself (three large hemispheric stroke, two basilar stroke, and one cerebral hemorrhage), and three likely died from non-stroke causes (two cardiopulmonary distress, one sepsis).

**Table 1:** Demographic and laboratory characteristics in all stroke, favorable and poor outcome groups

Characteristic	All (number = 175)	Favorable (number 111)	Poor (number = 64)	*P*
Age (years old)	66 ± 11	64 ± 11	69 ± 10	0.002
Sex (male, %)	95 (54.3)	56 (50.5)	39 (60.9)	0.21
Initial NIHSS score	5 (3 to 10)	4 (2 to 5)	12 (7 to 16)	< 0.001**^a^**
72 hour-infarct volume (ml)**^b^**	5.9 (1.4 to 35.0)	3 (1 to 9)	55 (5 to 215)	< 0.001**^a^**
Sampling time (hours)	10 (7 to 16)	10 (7 to 17)	11 (8 to 15)	0.66**^a^**
Hypertension (%)	101 (57.7)	61 (55.0)	40 (62.5)	0.35
Diabetes mellitus (%)	53 (30.3)	36 (32.4)	17 (26.6)	0.50
Hyperlipidemia (%)	42 (24.0)	26 (23.4)	16 (25.0)	0.81
Heart failure (%)	17 (9.7)	8 (7.2)	9 (14.1)	0.19
Atrial fibrillation (%)	53 (30.3)	21 (18.9)	32 (50.0)	< 0.001
Smoking (%)	71 (40.6)	47 (42.3)	24 (37.5)	0.63
Previous statin use (%)	26 (14.9)	17 (15.3)	9 (14.1)	0.99
Systolic BP (mmHg)	134 ± 19	134 ± 19	133 ± 18	0.68
Diastolic BP (mmHg)	82 ± 12	81 ± 10	83 ± 14	0.21
Creatinine (mg/dl)	1.07 ± 0.56	1.04 ± 0.62	1.11 ± 0.42	0.46
Fasting glucose (mg/dl)	138 ± 55	135 ± 57	143 ± 53	0.37
LDL cholesterol (mg/dl)	115 ± 37	118 ± 29	109 ± 47	0.17
WBC (10**^3^**/μl)	7.8 ± 3.0	7.7 ± 2.7	9.2 ± 3.7	< 0.01
TOAST				< 0.01
LAA	52 (29.7)	35 (31.5)	17 (26.6)	
CE	49 (28.0)	25 (22.5)	24 (37.5)	
SVD	38 (21.7)	34 (30.6)	4 (6.3)	
Others	36 (20.6)	17 (15.3)	19 (29.7)	

^a^Statistical analysis conducted using the Mann-Whitney U test; ^b^hyperintense lesion on diffusion-weighted image 72 hours after stroke. BP, blood pressure; CE, cardioembolism; LAA, large-artery atherosclerosis; LDL, low density lipoprotein cholesterol; NIHSS, National Institutes of Health Stroke Scale; SVD, small vessel disease; TOAST, Trial of Org10172 in the Acute Stroke Treatment; WBC, white blood cell.

The median concentrations of individual blood markers are shown in Table 2. The median sampling time after stroke onset was 10 hours (IQR: 7 to 16). The poor outcome group had higher median blood concentrations of hFABP, S100B, IL-6, MMP-9, CRP, and D-dimer than the favorable outcome group. The blood concentrations of NSE, TNF-α and PAI-1 were not different between the two groups. As described above, most of the stroke patients displayed negative GFAP and VSNL-1 in the peripheral blood during this time period, indicating that these proteins are unlikely to be released from the brain to the bloodstream during this time window of stroke.

**Table 2:** Comparison of median concentrations of blood markers between favorable and poor outcome groups

	All (number = 175)	Favorable (number = 111)	Poor (number = 64)	*P* ^a^
Neuronal				
NSE (ng/ml)	6.5 (1.9 to 10.8)	5.6 (1.6 to 10.3)	7.7 (3.0 to 11.5)	0.14
Ngb (ng/ml)	0.0 (0.0 to 2.7)	0.0 (0.0 to 0.9)	0.3 (0.0 to 9.7)	< 0.001
hFABP (ng/ml)	10.0 (6.2 to 16.3)	8.4 (5.5 to 11.9)	16.3 (9.9 to 29.7)	< 0.001
VSNL-1 (%)**^b^**	12 (6.9)	7 (6.3)	5 (7.8)	0.95
Astroglial				
S100B (pg/ml)	31.9 (0.0 - 124.9)	20.0 (0 to 60.3)	135.2 (40.0 to 344.6)	< 0.001
GFAP (%)**^b^**	50 (28.6)	23 (20.7)	27 (42.2)	0.004
Inflammatory				
IL-6 (pg/ml)	4.6 (0.9 to 13.0)	2.2 (0.6 to 5.5)	12.1 (6.7 to 48.0)	< 0.001
MMP-9 (ng/ml)	65.4 (30.9 to 124.1)	45.7 (21.9 to 79.5)	120.5 (61.8 to 228.3)	< 0.001
TNF-α (pg/ml)	2.3 (0.0 to 8.9)	1.3 (0.0 to 8.0)	3.3 (0.0 to 10.4)	0.22
CRP (mg/dl)	0.26 (0.09 to 0.94)	0.19 (0.06 to 0.36)	0.87 (0.18 to 4.40)	< 0.001
Haemostatic				
PAI-1 (ng/ml)	11.1 (4.5 to 21.8)	10.3 (4.2 to 23.1)	11.5 (5.9 to 20.9)	0.57
D-dimer (ng/ml)	521 (368 to 1083)	466 (355 to 933)	938 (450 to 1629)	0.001

^a^Statistical analysis conducted by the Mann-Whitney U test; ^b^Categorical variables. Values in round brackets indicate interquartile range. CRP, C-reactive protein; GFAP, glial fibrillary acidic protein; hFABP, heart type fatty acid binding protein; MMP-9 matrix metalloproteinase-9; Ngb, neuroglobin; NSE, neuron-specific enolase; PAI-1, plasminogen activator inhibitor-1; VSNL-1, visinin-like protein 1.

The logistic regression analysis of individual blood markers after adjusting for age and initial NIHSS score revealed that plasma _log_IL-6 (adjusted OR: 1.75, 95% CI: 1.25 to 2.25, *P *= 0.001) and _log_hFABP (adjusted OR: 3.23, 95% CI: 1.44 to 7.27, *P *= 0.0045) reached a statistical significance (Table 3). The statistical significance of _log_IL-6 and _log_hFABP remained after further adjustment for 72-hour infarct volume (natural log-transformed) and Afib (_log_IL-6, OR: 1.74, 95% CI: 1.24 to 2.44, *P *= 0.003; _log_hFABP, OR: 3.23, 95% CI: 1.41 to 7.40, *P *= 0.001), time of stroke onset (_log_IL-6, OR: 1.74, 95% CI: 1.24 to 2.44, *P *= 0.001; _log_hFABP, OR: 3.23, 95% CI: 1.41 to 7.40, *P *= 0.005) and previous statin use (_log_IL-6, OR: 1.81, 95% CI: 1.27 to 2.57, *P *= 0.001; _log_hFABP, OR: 3.36, 95% CI: 1.46 to 7.47, *P *= 0.004). Adding the _log_IL-6 and _log_hFABP to the baseline clinical model improved the -2 log likelihood ratio (102.57 versus 123.01, *P *= 0.01) and goodness of fit (Hosmer-Lemeshow's test, *P *= 0.506). No multi-collinearity was found among the tested variables. In the Spearmann's correlation analysis, infarct volume was significantly correlated with IL-6 (*r *= 0.38, *P *< 0.001) but not hFABP (*r *= 0.126, *P *= 0.10).

**Table 3:** Multivariate logistic regression analysis of blood markers (natural log-transformed) between favorable and poor outcome groups

	OR (95% CI)	*P*	Adjusted OR^a^	*P*
Neuronal markers				
NSE	1.23 (0.89 - 1.68)	0.209	0.79 (0.49 - 1.26)	0.325
Ngb	1.73 (1.28 - 2.34)	0.0004	1.41 (0.89 - 2.24)	0.143
hFABP	4.90 (2.67 - 9.00)	< 0.0001	3.23 (1.44 - 7.27)	0.0045
VSNL-1**^b^**	1.26 (0.38 - 4.14)	0.704	0.87 (0.19 - 4.02)	0.854
Astroglial markers				
S100B	1.60 (1.39 - 1.92)	< 0.0001	1.20 (0.96 - 1.51)	0.115
GFAP**^b^**	2.79 (1.42 - 5.49)	0.003	1.15 (0.41 - 3.22)	0.791
Inflammatory markers				
IL-6	2.23 (1.68 - 2.95)	< 0.0001	1.75 (1.25 - 2.25)	0.001
TNF-α	1.22 (0.94 - 1.57)	0.134	1.35 (0.88 - 2.05)	0.166
CRP	1.63 (1.33 - 1.99)	< 0.0001	1.23 (0.94 - 1.62)	0.148
Blood-brain barrier marker				
MMP-9	2.19 (1.43 - 3.44)	0.005	1.65 (1.07 - 2.07)	0.014
Haemostatic markers				
PAI-1	1.05 (0.79 - 1.38)	0.748	1.07 (0.71 - 1.61)	0.751
D-dimer	2.49 (1.61 - 3.86)	< 0.0001	1.37 (0.74 - 2.57)	0.319

The favorable group is regarded as the reference (OR: 1.0). ^a^Adjustment of age and initial NIHSS score; ^b^categorical variables. CI, confidence interval; CRP, C-reactive protein; GFAP, glial fibrillary acidic protein; hFABP, heart type fatty acid binding protein; MMP-9 matrix metalloproteinase-9; Ngb, neuroglobin; NSE, neuron-specific enolase; OR, odds ratio; PAI-1, plasminogen activator inhibitor-1; VSNL-1, visinin-like protein 1.

**Figure 1:**
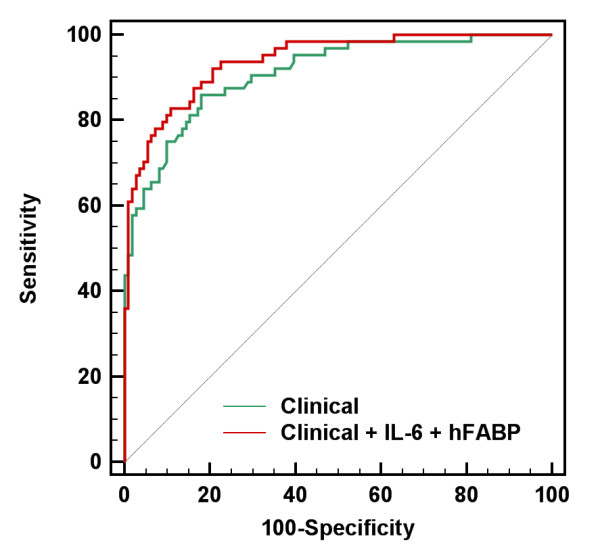
**Receiver operating characteristic (ROC) curve of the addition of the combination of _log_IL-6 and _log_hFABP to the clinical baseline model**.

In ROC curve analysis, the baseline clinical model showed good discriminating ability (AUC: 0.910) for stroke outcome. The addition of any single marker did not significantly improve the AUC value of the baseline clinical model. Adding a combination of _log_IL-6 and _log_hFABP to the baseline clinical model (AUC: 0.939, *P *< 0.03) significantly improved the discriminating ability of the baseline clinical model (Table 4 and Figure 1). The addition of other individual markers to the combined model of _log_IL-6, _log_hFABP and clinical model did not result in further improvement of AUC in ROC curve analysis (*P *≥ 0.05 by the DeLong method). We further calculated the in-sample NRI index and IDI of _log_IL-6 and _log_hFABP in each model (Table 4). Adding _log_IL-6 to the baseline clinical model significantly improved the NRI index (0.09, *P *= 0.04) but adding _log_hFABP to the baseline clinical model did not improve the NRI index. The addition of both _log_IL-6 and _log_hFABP further enhanced the NRI index of _log_IL-6 (0.18, *P *= 0.005). The in-sample reclassification table in the model with _log_IL-6 and _log_hFABP is presented in Additional file 2. In 64 patients with a poor outcome, seven were reclassified into higher risk categories, and only two were reclassified into lower risk categories. In 111 patients with a favorable outcome, only six were reclassified into higher risk categories, and 17 were reclassified into lower risk categories.

**Table 4:** Receiver operating characteristic (ROC) curve and in-sample reclassification analysis of the addition of each or the combination of IL-6 and hFABP (natural log-transformed) to the baseline clinical model for stroke outcome

	Discrimination		Reclassification			
		
	AUC (95% CI)	*P^b^*	NRI	*P^c^*	IDI	*P^d^*
Baseline clinical model**^a^**	0.910 (0.858 to 0.948)	-	-	-		
+ **_log_**IL-6	0.931 (0.883 to 0.964)	0.075	0.09	0.036	0.03	0.024
+ **_log_**hFABP	0.926 (0.877 to 0.960)	0.110	0.07	0.232	0.02	0.032
+ **_log_**IL-6 + **_log_**hFABP	0.939 (0.893 to 0.970)	0.032	0.18	0.005	0.06	0.001

^a^Model with age and initial NIHSS score; ^b^*P*-value for AUC using DeLong method with the baseline clinical model as a reference; ^c^*P*-value for net classification improvement with the baseline clinical model as a reference; ^d^*P*-value for integrated discrimination improvement with the baseline clinical model as a reference. AUC, area under curve; IDI, integrated discrimination improvement; _log_, natural log-transformed value; NRI, net reclassification improvement.

## Discussion

In the present study, we measured multiple blood markers that are plausibly related to the pathophysiology of ischemic stroke to predict functional outcome. The strengths of the present study include the prospective collection of stroke cases, measurement of multiple blood markers in a single study, exact diagnosis of stroke using DWI images in all cases and statistical analysis after adjusting for multiple confounding variables. We found that circulating IL-6 and hFABP levels showed an independent association with stroke prognosis after adjusting for clinical covariates. Moreover, the combination of these two markers showed a significant improvement of discrimination and reclassification for high and low risk of clinical outcome in stroke patients. Other blood markers failed to show a significant clinical value for the prediction of stroke outcome.

Previous studies demonstrated that IL-6 is one of the predictors of poor outcome in stroke [16-20]. Our finding of the strong association between IL-6 and poor stroke outcome is consistent with those of several studies using multiple blood markers in prospective stroke cohorts [4,5]. A systemic inflammatory response develops after brain damage during the acute phase of ischemic stroke. IL-6 is one of the most important mediators of the acute phase response to inflammation. The precise mechanism of the association between elevated IL-6 levels and poor clinical outcome is unclear. IL-6 up-regulates the expression of adhesion molecules, such as intercellular adhesion molecule-1 (ICAM-1), selectins and integrins on endothelial cells, leukocytes and platelets, during cerebral ischemia, which leads to secondary neuronal damage [21]. IL-6 also increases the levels of fibrinogen and von Willebrand factor, resulting in hypercoagulant status [22]. IL-6 expression is increased in the brain following ischemia, and damaged neurons may contribute to increased IL-6 levels [23]. Although the source of circulating IL-6 during the acute phase of stroke is still unknown, the early increase in IL-6 and its correlation with poor outcome support the role of acute inflammation in the pathophysiology of stroke.

Using a blood IL-6, we did not find any improvement in the prognostic performance of the clinical model for stroke outcome despite the close pathophysiological relationship between IL-6 and cerebral ischemia. Our results are consistent with previous studies which showed that IL-6 lacks clinical usefulness in the discrimination and reclassification between favorable and poor outcome groups of stroke patients [4,5]. In our data, the combined use of IL-6 and hFABP significantly improved the discriminating ability of the clinical model poor stroke outcome. The addition of IL-6 and hFABP significantly reclassified patients at low- and high-risk for poor outcome across the clinically relevant threshold. hFABP is a small cytoplasmic protein (15 kDa) that is involved in active fatty acid metabolism in the myocardium and neuronal cell bodies in the central nervous system [24]. Plasma hFABP level is known to be a sensitive biomarker of acute coronary ischemia [25,26]. In ischemic stroke, plasma hFABP levels were found to be elevated in several case-control studies [27-29]. Plasma hFABP levels six hours after stroke were associated with early neurological severity (NIHSS score at 10 days) and long-term outcome (mRS at three months) [28,29]. The precise mechanism of the association between blood hFABP and stroke outcome is unclear. One plausible explanation is that hFABP is released from the brain following cerebral ischemia. Proteomic analysis showed that hFABP levels in cerebrospinal fluid were elevated in deceased stroke patients [27]. However, we did not find a significant correlation between infarct volume and hFABP, indicating the extracerebral origin of hFABP release following stroke. A second explanation is that the elevation of blood hFABP reflects acute cardiovascular dysfunction associated with neuronal injury [30], which is associated with poor outcome. A third explanation is that hFABP is elevated in other conditions, such as heart failure [31], sepsis [32], pulmonary embolism [33], and metabolic syndrome [34], which may be an underlying substrate for poor stroke outcome. Further studies are required to clarify the origin of hFABP release during the acute phase of stroke and its predictive value for stroke outcome.

Neuro-astroglial markers are candidate stroke biomarkers because they are hypothetically more specific to brain injury than inflammatory and haemostatic markers. Several case-control studies showed that blood levels of S100B, NSE and GFAP are elevated in acute stroke, and are associated with poor stroke outcome [7-9]. We evaluated the blood profiling of several neuro-astroglial markers, including candidate neuronal markers (VSNL-1 and Ngb) that have not been tested in human stroke patients [35,36]. None of the tested markers, with the exception of hFABP, reached statistical significance. The lack of an association between blood levels and stroke outcome in the present study is attributable to their slow release kinetics from damaged tissue to peripheral blood (that is, their peak plasma levels were reached at 48 to 72 hours after stroke onset) [8,28].

The limitations of the present study should be addressed. First, we did not perform serial measurements of the blood markers. The inflammatory process and release of neuro-astroglial proteins are prominent for several days after a stroke. Therefore, the measurement of blood markers within 24 hours after stroke onset weakens the potential application. The aim of our study is to search for panel markers within 24 hours of stroke onset for prediction of stroke outcome, as prediction of stroke outcome is required as soon as possible considering the high mortality and morbidity of stroke. We also excluded patients with their onset within six hours because most cases during this time period need thrombolytic therapy, which may confound the clinical outcome of cerebral infarction, and because our experience has shown that most of the tested biomarkers (especially neuro- and astroglial proteins) are not elevated within six hours after symptom onset. Second, it is unknown whether the elevation of blood markers represents a consequence or a pre-morbid status of stroke, as inflammatory and haemostatic markers are elevated in non-cerebral conditions, such as systemic infection, cancer or metabolic diseases. Further studies that involve serial measurement of blood marker levels are required to resolve this issue. Third, the sample size of our study was small. Our results should be interpreted with caution, and external validation is required to be generalized in clinical practice. Fourth, we could not examine the candidate blood markers for prediction of stroke outcome in the previous studies, including brain natriuretic peptide (BNP or NT-proBNP) [4], midregional pro-atrial natriuretic peptide (MR-proANP) [37], lipoprotein-associated phospholipase A2 (LpPlA2) [38], or copeptin [39]. Further studies that expand the panel of blood markers screened are required.

## Conclusions

We found that blood IL-6 and hFABP levels are associated with poor clinical outcome in patients with ischemic stroke. Single markers did not enhance the discriminating ability of the clinical predictors for stroke outcome. The addition of the IL-6 and hFABP levels improved the prognostic performance of the clinical predictors for stroke outcome. Further studies in a large-scale prospective cohort are required to determine whether these blood proteins are clinically useful in routine clinical practice for prediction of those at high-risk of a poor clinical outcome, who need more intensive therapeutic strategies.

## Key messages

• Combination of multiple blood markers that involve different biological pathways may enhance the predictability of clinical parameters for stroke outcome.

• Blood IL-6 and hFABP levels were independently associated with the functional outcome at three months in patients with ischemic stroke.

• Combination of blood markers including IL-6 and hFABP improved the predictive value of clinical predictors for stroke outcome.

## Abbreviations

3m-mRS: modified Rankin score at three months; Afib: atrial fibrillation; AUC: area under the curve; CRP: C-reactive protein; DBP: diastolic blood pressure; DWI: diffuse weighted image; ELISA: enzyme-linked immunosorbent assay; GFAP: glial fibrillary acidic protein; hFABP: heart-type fatty acid binding protein; IDI: integrated discrimination index; IL-6: interleukin-6; MMP-9: matrix metalloproteinase-9; MRI: magnetic resonance imaging; Ngb: neuroglobin; NIHSS: National Institutes of Health Stroke Scale; NRI: net reclassification improvement; NSE: neuron-specific enolase; OR: odds ratio; PAI-1: plasminogen activator inhibitor-1; ROC: receiver operating characteristic; SBP: systolic blood pressure; TNF-α: tumor necrosis factor-α; TOAST: Trial of Org10172 in Acute Stroke Treatment; VSNL-1: visinin-like portein-1; WBC: white blood cells.

## Competing interests

The authors declare that they have no competing interests.

## Authors' contributions

SYP, SHO and OJK developed the study concept and design, and wrote the draft. JKK and JS performed the acquisition of data and analysis. JK, DAS and SHO conducted the interpretation of the data. JS, JKK, DAS and SHO made substantial revision of the report. All authors read and approved the final manuscript for publication.

## Supplementary Material

Additional file 1 **Methods for individual blood markers**. Click here for file

Additional file 2 **Reclassification table for favorable and poor stroke outcome**. Click here for file
