# Supplementary material for: Predictive value of circulating interleukin-6 and heart-type fatty acid binding protein for three months clinical outcome in acute cerebral infarction: multiple blood markers profiling study
Source: Crit Care. 2013 Mar 16;17(2):R45. doi: 10.1186/cc12564 (PMC3672476; doi:10.1186/cc12564)
Supplement: Additional file 1 — Methods for individual blood markers. [file cc12564-S1.DOC]

Additional file 1

Methods for individual blood markers

Whole blood (10cc) was drawn on arrival at the emergency department using the tube containing ethylenediaminetetraacetic acid (EDTA), heparin or citrate. The samples were immediately delivered to the laboratory. Plasma and serum were quickly prepared from whole blood by centrifugation at 3000 g for 15 min at room temperature. The plasma and serum were carefully transferred into appropriately labeled micro-centrifuge tubes by using a sterile transfer pipette tip. The samples were apportioned into 1 ml aliquots from 5 to 7 vials and stored at –80 ˚C for later analysis. Detailed methods for individual blood marker are described below:

1) Neuron-specific enolase (NSE, ng/ml)

Whole blood was drawn using the EDTA tube, and serum concentration of NSE was measured using a quantitative sandwich ELISA kit (IBL-America, MN) following the manufacturer's protocols. In brief, streptavidin microplate was washed with washing solution and 25μl of calibrators and samples was added in each well. 100μl of HRP anti-NSE monoclonal antibody mixed with biotin anti-NSE monoclonal antibody was added in each well and the wells were incubated for 1 hour shaking 300rpm at room temperature. Following washing with wash solution, 100μl of TMB-HRP was added in each well and the wells were incubated for 30minute shaking at room temperature. After 100 μl of stop solution was added in each well, the wells were incubated for 1minute shaking at room temperature. The reaction was stopped and absorbance of the resulting yellow product is measured at 450nm. Intra- and inter-assay CV were < 5% and <5%, respectively (manufacturer’s information). The MDD of the kit is typically 1ng/ml (manufacturer’s information). The median concentration of serum NSE in non-stroke subjects (n=90) in our laboratory was 4.2 ng/ml (IQR: 2.1 – 6.9).

2) Neuroglobin (NGB, ng/ml)

Whole blood was drawn using the EDTA tube, and plasma concentration of NGB was measured using a quantitative sandwich ELISA kit (CUSABIO biotech Co., Ltd, Wuhan, China) following the manufacturer's protocols. In brief, 100μl of standards, and samples was added the pre-coated microtiter plate with an antibody specific to NGB and the wells were incubated for 2 hours at 37˚C. 100μl of biotin-conjugated antibody preparation specific for NGB was added to the wells and the wells incubated for 1 hour at 37˚C. After washed three times, the wells were added with 100μl of HRP-avidin (Avidin-conjugated to Horseradish Peroxidase) working solution and incubated then for 1hour at 37˚C. Following washing five times with wash solution, 90μl of TMB solution was added to each well and the wells were incubated for 20minutes at 37˚C in the dark. The reaction was stopped by adding 50μl of stop solution and the intensity of the color is measured at 450nm. The concentration of NGB in the samples was then determined by comparing the O.D. of the samples to the standard curve. Intra- and inter-assay CV were < 8% and < 10%, respectively (manufacturer’s information). The MDD of the kit is typically 0.47ng/ml (manufacturer’s information). The median concentration of plasma NGB in non-stroke subjects (n=90) in our laboratory was 0.5 ng/ml (IQR: 0.0-3.7).

3) Heart-type fatty acid binding protein (hFABP, ng/ml)

Whole blood was drawn using the EDTA tube, and plasma concentration of S100B was measured using a solid-phase ELISA based on the sandwich principle (Hycult Biotechnology, Uden, The Netherlands). In brief, after adding 50μl of diluted tracer-peroxidase second antibody to each well coated with antibody recognizing human hFABP, 50μl of standards and diluted plasma samples were transferred into appropriate wells. The wells were incubated for 60min at room temperature and washed 4 times with 200μl of washing buffer. 100μl of tetramethylbenzidine (TMB) substrate was added to each well and the wells were incubated for 15minutes avoiding direct sunlight. The enzyme reaction was stopped by the addition of 100μl oxalic acid stop solution and absorbance of the resulting yellow product is measured at 450nm with a spectrophotometer. The concentration of hFABP in the samples was then determined by comparing the O.D. of the samples to the standard curve. Intra-assay and inter-assay CV were 5.6% and 8.1%, respectively (manufacturer’s information). The MDD of the kit is typically 0.1 ng/ml (manufacturer’s information). The median concentration of plasma hFABP in non-stroke subjects (n=90) in our laboratory was 6.7 ng/ml (IQR: 5.0-9.6).

4) Visinin-like protein -1 (VSNL-1, ng/ml)

Whole blood was drawn using the EDTA tube, and plasma concentration of VSNL-1 was measured using a quantitative sandwich ELISA kit (BioVendor, Candler, USA). In brief, 100μl of standards, control and diluted samples was incubated in microplate wells pre-coated with polyclonal anti-human VSNL -1 antibody at room temperature, shaking at ca.300rpm on orbital microplate shaker. After 60minutes incubation and washing 5-times with wash solution, biotin labeled polyclonal anti-human VSNL-1 antibody was added and incubated with captured VSNL-1 for 60 minutes. After washing 5-times, streptavidin-HRP conjugate was added the wells, shaking at ca.300rpm on orbital microplate shaker at room temperature. After 60minutes incubation and the last washing step, the remaining conjugated was allowed to react with the substrate solution (TMB). The reaction was stopped by addition of acidic of proportional to the concentration. The concentration of VSNL-1 in the samples was then determined by comparing the O.D. of the samples to the standard curve. Intra- and inter-assay CV were 5.33% and 3.22%, respectively (manufacturer’s information). The MDD of the kit is typically 0.027 ng/ml (manufacturer’s information). The positive detection rate of plasma GFAP in non-stroke subjects (n=90) in our laboratory was 11.1% (n=10).

5) S100B (pg/ml)

Whole blood was drawn using the heparin tube, and plasma concentration of S100B were measured using sandwich enzyme-linked immunosorbent assay immunoassay (ELISA) kit (Abnova, Taipei, Taiwan) following the manufacturer's protocols. In brief, 100μl of diluted standards, quality controls, dilution buffer and diluted samples was incubated into pre-coated microplate wells with polyclonal anti-cow S100B antibody at room temperature for 120minutes, shaking at ca. 300rpm on an orbital microplate shaker. After washing the wells 3-times with washing solution (0.35ml per well), the wells were incubated with biotin labeled monoclonal anti-human S100B antibody at room temperature for 60minutes, shaking at ca. 300rpm on an orbital microplate shaker. After another washing 5-times, 100 μl streptoavidin-HRP conjugate was added into the wells and the wells were incubated at room temperature for 30minutes, shaking at ca. 300rpm on an orbital microplate shaker. After the last washing, the wells were incubated with the 100μl substrate solution (TMB) for 15minutes, covered with aluminum foil. The reaction is stopped by addition of 100μl stop solution and absorbance of the resulting yellow product is measured at 450nm. Intra- and inter-assay coefficients of variation (CV) were 3.25% and 7.65%, respectively (manufacturer’s information). The minimum detectable dose (MDD) of the kit is 15 pg/ml (manufacturer’s information). The median concentration of plasma S100B in non-stroke subjects (n=90) in our laboratory was 2.3 pg/ml (interquartile range [IQR]: 0.0 – 20.6).

6) Glial fibrillary acidic protein (GFAP, ng/ml)

Whole blood was drawn using the EDTA tube, and plasma concentration of GFAP was measured using a quantitative sandwich ELISA kit (Abnova, Taipei, Taiwan) following the manufacturer's protocols. In brief, 100μl of diluted standards, quality controls, dilution buffer and diluted samples were incubated into pre-coated microplate wells with polyclonal anti-human GFAP antibody at room temperature for 120minutes, shaking at ca. 300rpm on an orbital microplate shaker. After washing the wells 3-times with washing solution (0.35ml per well), the wells were incubated with biotin labeled monoclonal anti-human GFAP antibody at room temperature for 60minutes, shaking at ca. 300rpm on an orbital microplate shaker. After another washing 3-times, 100μl streptoavidin-HRP conjugate was added into the wells and the wells were incubated at room temperature for 60minutes, shaking at ca. 300rpm on an orbital microplate shaker. After the last washing, the wells were incubated with the 100μl substrate solution (TMB) for 15minutes, covered with aluminum foil. The reaction was stopped by addition of 100μl stop solution and absorbance of the resulting yellow product is measured at 450nm. Intra- and inter-assay CV were 5.1% and 5.63%, respectively (manufacturer’s information). The MDD of the kit is typically 0.045ng/ml (manufacturer’s information). The positive detection rate of plasma GFAP in non-stroke subjects (n=90) in our laboratory was 15.6% (n=14).

7) Interleukin-6 (IL-6, pg/ml)

Whole blood was drawn using the EDTA tube, and plasma concentration of IL-6 was measured using a quantitative sandwich ELISA kit (IL-6 Quantikine, R&D Systems, MN). In brief, 100μl of assay diluent was added and 100μl of standards, control and samples was incubated in microplate wells pre-coated with monoclonal anti-human IL-6 antibody for 2hours at room temperature. After washing away any unbound substances, an enzyme-linked polyclonal antibody specific for IL-6, IL-6 conjugate, was added to the each wells and the wells were incubated 2hours at room temperature. Following a wash to remove any unbound antibody-enzyme reagent, a substrate solution is added to the wells and color develops in proportion to the amount of IL-6 bound in the initial step. The color development is stopped and the intensity of the color is measured at 450nm. The concentration of IL-6 in the samples was then determined by comparing the O.D. of the samples to the standard curve. Intra- and inter-assay CV were 2.6% and 4.5%, respectively (manufacturer’s information). The MDD of the kit is typically 0.70 pg/ml (manufacturer’s information). The median concentration of plasma IL-6 in non-stroke subjects (n=90) in our laboratory was 1.2 pg/ml (IQR: 0.0 - 2.4).

8) Matrix-metalloproteinase-9 (MMP-9, ng/ml)

Whole blood was drawn using the citrate tube, and plasma concentration of MMP-9 was measured using a commercially available ELISA kit (e-bioscience, Abingdon, UK). In brief, 100μl of diluted standards and samples was added and 50μl of biotin-conjugate was added in anti human MMP-9 monoclonal antibody pre-coated microplate. After incubation for 2hours shaking 100rpm on orbital microplate shaker at room temperature, the wells were washed 4 times. 100μl of diluted streptavidin-HRP was added in the wells and the wells were incubated for 1hour shaking 100rpm on orbital microplate shaker at room temperature. Following a wash to removes any unbound antibody-enzyme reagent, 100μl of TMB substrate solution was added in the wells for 10minutes. The remaining conjugated was allowed to react with the substrate solution (TMB). The reaction was stopped by addition of 100μl stop solution and absorbance of the resulting yellow product is measured at 450nm. The concentration of MMP-9 in the samples was then determined by comparing the O.D. of the samples to the standard curve. The measurement range of human MMP-9 starts at 0.23ng with an upper limit of quantification of 15ng/ml. After pre-dilution 1:10 with the assay buffer (1x), plasma samples were diluted 1: 20 for MMP-9. The measured concentration of sample from standard curve was calculated by dilution factor, because samples had been diluted prior to the assay. Intra-assay and inter-assay CV were 7.3% and 10.2%, respectively (manufacturer’s information). The MDD of the kit is typically 0.05 ng/ml (manufacturer’s information). The median concentration of plasma MMP-9 in non-stroke subjects (n=90) in our laboratory was 33.8 ng/ml (IQR: 15.4 – 60.8).

9) Tumor necrosis factor-alpha (TNF-α, pg/ml)

Whole blood was drawn using the EDTA tube, and plasma concentration of TNF-α was measured using a quantitative sandwich ELISA kit (R&D Systems, Minneapolis, USA). In brief, after adding 50μl of buffered protein base with preservative, 200μl of standards and samples were incubated in TNF-α monoclonal antibody pre-coated microplate. After 2hours, the wells were washed 4-times with wash solution. Horseradish peroxidase conjugated polyclonal anti-human TNF-α antibody was added in the wells and the wells were incubated for 2hours at room temperature. Following a wash to removes any unbound antibody-enzyme reagent, 200μl of substrate solution was added to the wells and color development was stopped by stop solution. The intensity of the color was measured at 450nm within 30minutes. The concentration of TNF-α in the samples was then determined by comparing the O.D. of the samples to the standard curve. Intra-assay and inter-assay CV were 4.7% and 5.8%, respectively (manufacturer’s information). The MDD of the kit is typically 1.6 pg/ml (manufacturer’s information). The median concentration of plasma TNF-α in non-stroke subjects (n=90) in our core laboratory was 2.6 pg/ml (IQR: 0.0 – 7.4).

10) C-reactive protein (CRP, mg/dl)

Whole blood was drawn using the EDTA tube, and the test was performed in the core laboratory of our hospital. In brief, the serum concentration of CRP was measured using a particle-enhanced immunoturbidimetric assay with CRP latex reagent (Roche Diagnostics, Mannheim, Germany) following the manufacturer’s protocol. The results were automatically calculated by the Hitachi 7600 autoanalyzer (Hitachi High Technologies co., Tokyo, JAPAN). The intra- and inter-assay CV were 5.5% and 6.5%, respectively (manufacturer’s information). The MDD of the kit is typically 0.03 ng/ml (manufacturer’s information). The normal range of CRP in normal healthy controls in our core laboratory was less than 0.3 mg/dl. (manufacturer’s information)

11) Plasminogen activator inhibitor-1 (PAI-1, ng/ml)

Whole blood was drawn using the citrate tube, and plasma concentration of PAI-1 was measured using a commercially available active PAI-1 functional assay kit (Innovative Research, Novi, MI, USA). In brief, adding 100 μl of diluted standards and samples, the urokinase coated microtiter wells were incubated for 30minutes shaking 300rpm on orbital microplate shaker at room temperature. After washing 3 times, 100μl of BSA mixed PAI-1 primary antibody was added in the wells and the wells were incubated for 30minutes, shaking at ca.300rpm on orbital microplate shaker at room temperature. Following a washing, 100μl of BSA mixed PAI-1 conjugated secondary antibody was added the wells were incubated for 30minutes, shaking at ca.300rpm on orbital microplate shaker at room temperature. After washed 3 times, 100μl of tetramethylbenzidine (TMB) substrate was added to each well and the wells were incubated for 10minutes avoiding direct sunlight. The enzyme reaction was stopped by the addition of 50μl stop solution and absorbance of the resulting yellow product is measured at 450nm with a spectrophotometer. The concentration of PAI-1 in the samples was then determined by comparing the O.D. of the samples to the standard curve. Correlation coefficient was 0.996 between the standard samples (manufacturer’s information). The MDD of the kit is typically 0.181ng/ml (manufacturer’s information). The median concentration of plasma PAI-1 in non-stroke subjects (n=90) in our laboratory was 6.6 ng/ml (IQR: 3.4 – 16.0).

12) D-dimer (ng/ml)

Whole blood was drawn using the citrate tube, and the test was performed in the core laboratory of our hospital. With VIDAS D-dimer reagent (VIDAS® D-dimer exclusion^TM^ II, bioMérieux, France), fibrin degradation products are measured with a two-step enzyme-linked fluorescent assay (ELFA), which combines the ELISA method with a final detection in fluorescence. The test is fully automated on the bioMérieux immunoassay system VIDAS® D-dimer exclusion^TM^ II, bioMérieux, France). The results are automatically calculated by the instrument in relation to calibration curve stored in memory and a report is then printed. The intra- and inter-assay CV were 2% and 6.1%, respectively (manufacturer’s information). The MDD of the kit is typically 45 ng/ml (manufacturer’s information). The normal range of plasma D-dimer in healthy controls was less than 500 ng/ml.
