# Supplementary material for: Predictive value of circulating interleukin-6 and heart-type fatty acid binding protein for three months clinical outcome in acute cerebral infarction: multiple blood markers profiling study
Source: Crit Care. 2013 Mar 16;17(2):R45. doi: 10.1186/cc12564 (PMC3672476; doi:10.1186/cc12564)
Supplement: Additional file 2 — Reclassification table for favorable and poor stroke outcome. [file cc12564-S2.DOC]

Additional file 2

**Reclassification table for favorable and poor stroke outcome**

| Adding IL-6 and hFABP model | | | | | | |
| --- | --- | --- | --- | --- | --- | --- |
| Poor outcome | | < 10 % | 10 – 49 % | 50 – 89 % | ≥ 90 % | Sum |
| Baseline clinical model^a^ | < 10 % | 1 | 1 | 0 | 0 | 2 |
|  | 10 – 49 % | 0 | 14 | 3 | 1 | 18 |
|  | 50 – 89 % | 0 | 2 | 11 | 2 | 15 |
|  | ≥ 90 % | 0 | 0 | 0 | 29 | 29 |
|  | Sum | 1 | 17 | 14 | 32 | 64 |
| Adding IL-6 and hFABP model | | | | | | |
| Favorable outcome | | < 10 % | 10 – 49 % | 50 – 89 % | ≥ 90 % | Sum |
| Baseline clinical model^a^ | < 10 % | 49 | 4 | 1 | 0 | 54 |
|  | 10 – 49 % | 16 | 31 | 1 | 0 | 48 |
|  | 50 – 89 % | 0 | 1 | 7 | 0 | 8 |
|  | ≥ 90 % | 0 | 0 | 0 | 1 | 1 |
|  | Sum | 65 | 36 | 9 | 1 | 111 |

^a^ Logistic model with age and initial NIHSS score.

IL-6: interleukin-6, hFABP: heart-type fatty acid binding protein
